# Integrated multi-omics analysis identifies potential therapeutic targets for Alzheimer’s disease

**DOI:** 10.4103/NRR.NRR-D-25-00493

**Published:** 2025-09-29

**Authors:** Hongli Li, Jin Kang, Zilin Liang, Xiaowei Wang, Lemei Zhu, Hanfen Tang, Weijun Peng

**Affiliations:** 1 Department of Integrated Traditional Chinese & Western Medicine, The Second Xiangya Hospital, Central South University, Changsha, Hunan Province, China; 2 Department of Rheumatology and Immunology, The Second Xiangya Hospital of Central South University, Changsha, Hunan Province, China; 3 School of Integrated Chinese and Western Medicine, Hunan University of Chinese Medicine, Changsha, Hunan Province, China; 4 Department of Pathology, The Second Xiangya Hospital, Central South University, Changsha, Hunan Province, China; 5 Academician Workstation, Changsha Medical University, Changsha, Hunan Province, China; 6 Department of Nutrition, Second Xiangya Hospital, Central South University, Changsha, Hunan Province, China

**Keywords:** amyloid-β, amyloid precursor protein/presenilin 1, astrocyte, endocytosis, glial fibrillary acidic protein, multi-omics, nerve regeneration, post-transcriptional modification, post-translational modification, RTN4

## Abstract

Because the pathogenesis of Alzheimer’s disease is multifactorial and complex, integrated multi-level omics analysis is essential to comprehensively elucidate its molecular alterations. We therefore utilized the well-established amyloid precursor protein/presenilin 1 mouse model to carry out an integrated multi-omics study using transcriptomic, proteomic, N^6^-methyladenosine epitranscriptomic, and phosphoproteomic analyses. The results revealed substantial molecular alterations across multiple biological dimensions and the alteration in the expression of several key genes, such as *GFAP*, *APP*, and *RTN4*, in a mouse model of Alzheimer’s disease. The pronounced elevation of RTN4 in reactive astrocytes is indicative of its involvement in Alzheimer’s disease pathogenesis. Furthermore, we identified dysregulation of pathways related to endocytosis, highlighting the critical role of this process in disease progression. Our findings underscore the significant impact of post-transcriptional (N^6^-methyladenosine methylation) and post-translational (phosphorylation) protein modifications, which have been underrepresented in Alzheimer’s disease research. The significant contribution made by this study is the integrated, multi-level omics analysis that we carried out to investigate the complex biological changes that occur in Alzheimer’s disease. Our findings provide novel insights into Alzheimer’s disease pathogenesis and suggest potential therapeutic targets, such as RTN4.

## Graphical abstract

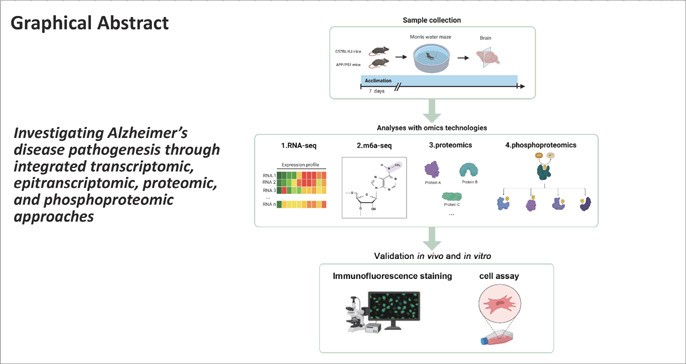

## Introduction

Alzheimer’s disease (AD), the most common form of neurodegeneration, is characterized by progressive cognitive decline, as well as the accumulation of amyloid-β (Aβ) plaques and neurofibrillary tangles (Yeapuri et al., 2023; Korczyn and Grinberg, 2024; Shanks et al., 2024; Zhang et al., 2024). The precise molecular mechanisms underlying AD are not fully understood (Blumenfeld et al., 2024), and currently there are no curative treatments available (Wagemann et al., 2024), suggesting that there is an urgent need for new therapies.

Recent developments in high-throughput omics have improved our understanding of the molecular alterations associated with AD by enabling detailed analysis at the transcriptomic and proteomic levels (Johnson et al., 2018; Bai et al., 2020). Nevertheless, traditional single-layer omics approaches often overlook the intricate regulatory relationships that govern gene expression. As per the central dogma of molecular biology, the usual flow of information from DNA to RNA to protein suggests a correlation between transcript and protein abundance (Yang et al., 2020; Diercks et al., 2021; Wingo et al., 2021). However, it is widely recognized that regulation at the transcriptional, post-transcriptional, and translational levels often creates large discrepancies between mRNA transcript levels and their respective protein expression levels (Buccitelli and Selbach, 2020; Slobodyanyuk et al., 2024). Consequently, multi-omics integration is necessary to achieve a better comprehensive understanding of AD pathology.

Several recent integrative studies have shown that multi-omics methods can elucidate the complex molecular networks involved in AD pathogenesis (Hampel et al., 2021; Lim et al., 2024; Rahimzadeh et al., 2024). Circular RNA (circRNA)-related competing endogenous RNA (ceRNA) networks may affect dendritic growth and memory in amyloid precursor protein/presenilin 1 (APP/PS1) mice (Ma et al., 2019). Using transcriptomic, proteomic and epigenomic profiling of postmortem human brains, Nativio et al. (2020) found that AD involves significant reconfiguration of the epigenome, which dysregulates transcription and chromatin–gene feedback loops. These studies underscore the advantages of multi-omics approaches in revealing previously unrecognized aspects of AD biology. Still, current multi-omics studies primarily focus on the transcriptomic and proteomic landscapes, while RNA methylation and post-translational modulations such as phosphorylation have received less attention.

Increasing evidence shows that N^6^-methyladenosine (m^6^A) and protein phosphorylation are important post-transcriptional and post-translational regulatory mechanisms that might participate in AD pathogenesis (Singh et al., 2023; Knight et al., 2024). Phosphorylation of various proteins, especially Tau, is associated with AD progression. Tau phosphorylation causes neuronal dysfunction and subsequent neurodegeneration (Capuano et al., 2025). Likewise, m^6^A RNA modifications regulate important RNA metabolic processes, such as stability, translation efficiency, splicing, and nuclear export (Kumari et al., 2022; Li et al., 2022b; Zhang and Wang, 2023). Disruption of m^6^A methylation has been linked to impaired neurogenesis, memory loss, and inhibition of neuronal regeneration in different neurodegenerative contexts (Jiang et al., 2021; Shafik et al., 2021; Castro-Hernández et al., 2023; Cheng et al., 2023; Rahimzadeh et al., 2024). Thus, combined investigation of these modifications via multi-omics system analysis is expected to provide more comprehensive insight into AD biology.

In this study, we systematically investigated AD-related molecular alterations by conducting multi-omics analyses of APP/PS1 transgenic mice integrating transcriptomics, proteomics, m^6^A epitranscriptomics, and phosphoproteomics. The key findings from these comprehensive analyses were validated in the human SVGP12 astrocyte cell line.

## Methods

### Animals

Male C57BL/6J (wild type, WT) and APP/PS1 mice, aged 9 months, weighed 35 ± 1 g, were obtained from SiPeifu Biotechnology (Beijing, China, license No. SCXK (Jing) 2019-0010) and allowed to acclimate to the housing conditions for 2 weeks before any experiments were performed. The mice were housed under specific pathogen–free conditions, with a controlled 12/12-hour light/dark cycle, an ambient temperature maintained at 21–23°C, and a relative humidity ranging from 40% to 60%. They had free access to standard diet and water. All animal experiments were reviewed and approved by the Animal Ethics Committee of Changsha Medical University (approval No. D2023020) on November 10, 2023. All experiments were designed and reported according to the Animal Research: Reporting of *In Vivo* Experiments (ARRIVE) guidelines (Percie du Sert et al., 2020).

### Morris water maze test

The Morris water maze (MWM) test was utilized to assess spatial learning and memory, as described in earlier studies (Li et al., 2022a, 2025b). The MWM setup (Xinruan, Shanghai, China, XR-XM101) included a circular pool with a diameter of 1.6 m. The water was maintained at a controlled temperature and was divided into four quadrants of equal size. A hidden platform was located about 1–2 cm below the water in the middle of the fourth quadrant. For 5 days (acquisition phase), the mice were trained by placing each mouse in a different starting quadrant and allowing them 1 minute to find the hidden platform. The time taken to reach the platform was recorded to evaluate learning. If mice were unable to find the platform within a specified time frame, they were guided to the platform manually and allowed to remain there for about 20 seconds for memory reinforcement. On day 6, for the probe trial, the hidden platform was removed to evaluate memory retention. On day 6 (test phase), each animal was placed in the pool for 60 seconds. Numerous parameters were recorded and analyzed, including the latency to reaching the original platform location and the number of platform area crossings.

### Immunofluorescence and immunohistochemistry staining

Brain tissues from both WT and APP/PS1 mice were processed following established protocols. First, the mice were sacrificed with an overdose of pentobarbitone sodium (100 mg/kg body weight; FWD Chemicals Ltd., Shanghai, China) via intraperitoneal injection, and their brains were extracted and fixed in 4% paraformaldehyde for 24 hours at 4°C. Following fixation, the tissues were dehydrated in a gradient ethanol series (70%, 80%, 95%, and 100%). Paraffin-embedded brain samples were then sectioned at 5-µm thickness using a Leica RM2235 microtome (Leica Biosystems, Wetzlar, Germany). The sections were mounted onto glass slides and stored at room temperature until further processing. Antigen retrieval was performed to reveal antigenic sites which may have been masked during tissue fixation by placing the slides in pre-warmed citric acid buffer (pH 6.0) at 95°C for 1–2 hours to restore epitope accessibility. The slides were allowed to cool to room temperature and then washed for 10 minutes in phosphate-buffered saline (PBS). To avoid interference with detection from endogenous peroxidase activity, the tissue sections were incubated with 0.3% hydrogen peroxide at 36–37°C for 10 minutes. Following this, the sections were washed with PBS for 10 minutes, then treated with 3% bovine serum albumin at 36–37°C for 30 minutes to minimize non-specific antibody binding. This blocking step prevents the antibody from binding to anything else apart from the target antigen.

For immunofluorescence staining, the brain tissue sections were washed, permeabilized, and blocked. First, the sections were incubated in permeabilization solution (0.2% Triton X-100 in PBS) for 30 minutes to facilitate antibody penetration. Then, the sections were blocked with 3% BSA for 30 minutes. Next, they were incubated with the following primary antibodies at 4°C for 12–15 hours: anti-RTN4 (rabbit polyclonal antibody, 1:200, Proteintech, Shanghai, China, Cat# 10740-1-AP, RRID: ab_2183598) and anti-GFAP (mouse monoclonal antibody, 1:500, Proteintech, Cat# 60190-1-Ig, RRID: AB_10838694). RTN4, also known as Nogo-A, is a neurite growth inhibitor associated with neurodegenerative diseases, while GFAP is a key marker of astrocyte reactivity (Kulczyńska-Przybik et al., 2021; Konno et al., 2024). After incubation with the primary antibodies, the tissue sections were incubated at room temperature (36°C) for 1 hour with suitable fluorophore-labeled secondary antibodies. The secondary antibodies used were Alexa Fluor 488 anti-rabbit (1:400, Life Technologies, Carlsbad, CA, USA, Cat# A21206, RRID: AB_141708) and Alexa Fluor 594 anti-mouse (1:1000, Abcam, Shanghai, China, Cat# ab97230, RRID: AB_10679334). Following incubation with the secondary antibodies, the sections were washed three times for 15 minutes each in PBS. The sections were then counterstained with 4′,6-diamidino-2-phenylindole (Servicebio, Wuhan, China, Cat# G1012) and rinsed in PBS, and coverslips were mounted using Prolong Gold antifade reagent (Thermo Fisher Scientific, Waltham, MA, USA) to preserve the fluorescence signal and prevent fading during imaging. Whole-brain immunofluorescence images were acquired using a 3DHISTECH slide scanning system (Budapest, Hungary).

For immunohistochemistry staining, parallel sections from the same tissue blocks were used to detect Aβ plaques. The brain sections were incubated with a primary antibody against Aβ (rabbit polyclonal antibody, 1:100, Servicebio, Cat# GB111197, RRID: AB_3095308) at 4°C for 12–14 hours. Aβ plaques are primarily made up of aggregated Aβ peptides. These plaques are crucial in the development of AD (Tian et al., 2021). The brain sections were then washed with PBS for 10 minutes. Next, they were incubated with goat anti-rabbit IgG secondary antibody (1:1800, Abcam, Cat# ab205718, RRID: AB_2866496) conjugated to horseradish peroxidase (Servicebio) for 45 minutes at 37°C. Following washing with PBS, the sections were incubated with 3,3′-diaminobenzidine (Servicebio) to develop the horseradish peroxidase signal: when 3,3′-diaminobenzidine interacts with horseradish peroxidase, it produces a brown precipitate, which in this case indicated the presence of Aβ plaques in the tissue sections. To stop the reaction, the slides were rinsed in distilled water before counterstaining the tissue with hematoxylin for visualization of cell nuclei. The stained slides were then dehydrated in a graded alcohol series. The slides were imaged using a slide scanner (NanoZoomer S360, Hamamatsu Photonics, Shizuoka, Japan) to allow for high-resolution imaging of the Aβ plaque burden.

### RNA sequencing

RNA extraction and RNA sequencing were carried out as reported previously (Li et al., 2025a). Mouse brain tissues were homogenized using TRI Reagent® (Sigma-Aldrich, St. Louis, MO, USA), and total RNA was extracted utilizing a Direct-zol™ RNA MiniPrep kit following the manufacturer’s recommended protocol. An Agilent Bioanalyzer 2100 was used to assess RNA integrity and quality, and only samples with an RNA integrity value ≥ 9 were used. Next, mRNA was isolated from total RNA using NEBNext® Poly(A) mRNA Magnetic Isolation Module (New England Biolabs, Ipswich, MA, USA) as per manufacturer’s protocol. Libraries were constructed by ligating the isolated mRNA to adapters using an Illumina TruSeq® Stranded mRNA Library Preparation Kit (Illumina, San Diego, CA, USA). Excess adapters and primers were removed using AMPure XP Magnetic Beads (Beckman Coulter, Brea, CA, USA). The libraries were subjected to via eight amplification cycles using KAPA HiFi HotStart Ready Mix, followed by quantification using a KAPA Library Quantification Kit (Roche, Basel, Switzerland) and an Illumina Platform. Finally, these multiplexed libraries were combined at equal concentrations and sequenced on an Illumina NovaSeq 6000 system at about 30 million reads per sample.

Raw sequencing reads were aligned to the mouse reference genome (GRCm38, Ensembl) using the STAR aligner (version 2.7.3a; Cold Spring Harbor Laboratory, Cold Spring Harbor, NY, USA). Gene expression was quantified using RSEM (v1.3.1; University of Wisconsin-Madison, Madison, WI, USA), with transcript abundances normalized to transcripts per million. We then used DESeq2 (version 1.34.0; European Molecular Biology Laboratory, Heidelberg, Germany) to conduct a differential expression analysis. Differentially expressed genes were defined by the following criteria: fold change ≥ 1 and *P*-value < 0.05. The resulting data are presented as heatmaps, volcano plots, and principal component analysis (PCA) plots to illustrate expression differences between the APP/PS1 transgenic and WT mice.

### Methylated RNA immunoprecipitation sequencing

We performed methylated RNA immunoprecipitation sequencing (MeRIP-Seq) using a Magna MeRIP^TM^ m^6^A Kit (Merck Millipore, Burlington, MA, USA) according to the manufacturer’s protocol. Briefly, total RNA was initially treated with DNase I (to remove any potential genomic DNA contamination) and subsequently fragmented into **~**100-nucleotides fragments by incubating it with a dedicated RNA fragmentation reagent at 94°C for 5 minutes. The reaction was halted by adding a stop buffer, followed by ethanol precipitation to purify the fragmented RNA. For the immunoprecipitation assay, an anti-m6A antibody (12 µg per IP; rabbit polyclonal, Synaptic Systems, Göttingen, Germany, Cat# 202 003, RRID: AB_2279214) was mixed with protein A/G magnetic beads (50 µL) in immunoprecipitation buffer (150 mM NaCl, 0.1% NP-40, 10 mM Tris–HCl, pH 7.4) for 1 hour at room temperature. The prepared beads were incubated with the fragmented RNA samples (6 µg) at 4°C for 4–5 hours with continuous rotation. Next, the beads were washed with immunoprecipitation buffer to remove any nonspecifically bound materials, and treated with a high concentration of proteinase K to remove protein complexes from the immunoprecipitated RNA. Subsequently, RNA was extracted using phenol-chloroform, followed by ethanol precipitation. The purified RNA was either analyzed by quantitative polymerase chain reaction to confirm the presence of m^6^A modifications or directly used for library construction. For validation via MeRIP-quantitative polymerase chain reaction, primers were specifically designed based on predicted m^6^A modification sites within PD-L1 transcripts identified using the SRAMP bioinformatic prediction tool (http://www.cuilab.cn/sramp). The libraries were prepared using a SMARTer smRNA-Seq Kit (Clontech, Takara Bio, Kusatsu, Shiga, Japan), with a portion of the fragmented RNA used as the input. The libraries were sequenced on an Illumina HiSeq X Ten to generate the reads used for downstream epitranscriptomic analysis.

Total RNA was immunoprecipitated with an anti-m^6^A antibody to enrich for m^6^A-modified transcripts. Immunoprecipitated RNA and supernatant RNA (Sup) fractions were labeled with Cy5 and Cy3 fluorescent dyes, respectively, using a commercially available labeling kit (Arraystar, Rockville, MD, USA). The labeled cRNA samples were subsequently hybridized onto a customized Arraystar Human m^6^A Epitranscriptomic Microarray (8 × 60K) designed specifically for transcriptome-wide m^6^A detection. The arrays were scanned, and the data were visualized through heatmaps, volcano plots, and PCA analysis.

### Proteomics and phosphoproteomics analysis

Brain tissue was lysed in SDT buffer (4% sodium dodecyl sulfate, 100 mM Tris-HCl, 1 mM dithiothreitol, pH 7.6), and the total protein content was quantified by bicinchoninic acid assay. The proteins were filtered and then digested according to published procedures. The resulting peptides were purified using C18 solid-phase extraction cartridges (Empore^TM^ SPE Cartridges, Sigma, St. Louis, MO, USA), dried under vacuum, and reconstituted in 40 µL of 0.1% formic acid for subsequent analysis. For each sample (100 µg, *n* = 3), the peptides were labeled using a six-plex Tandem Mass Tag kit. The WT group was assigned tandem mass tags 126, 127, and 128, whereas tags 129, 130, and 131 were used for the APP/PS1 group. The labeled peptide samples were divided into two portions for proteomic and phosphoproteomic analyses and then separated with a high-pH reversed-phase fractionation kit. Selective phosphopeptide enrichment was carried out using a High-Select^TM^ Fe-NTA Kit (Thermo Fisher Scientific). The enriched phosphopeptides were lyophilized, then reconstituted with 20 µL 0.1% formic acid and subjected to liquid chromatography–tandem mass spectrometry (LC-MS/MS) analysis.

LC-MS/MS was carried out using a Q Exactive mass spectrometer interfaced with an Easy nano liquid chromatography system (Thermo Fisher Scientific). The phosphopeptides were separated on a 100 µm × 2 cm reverse-phase trap column, followed by an analytical column (75 µm × 10 cm), applying a 84% acetonitrile/0.1% formic acid gradient (300 nL/min). MS acquisition was performed in DDA mode. Spectral interpretation was performed using MASCOT (version 2.2; Matrix Science Ltd., London, UK) within Proteome Discoverer 2.4, querying NCBI_Crassostrea_gigas_65751_20210617.fasta. DEPs/DPP were defined by the following criteria: fold change > 1 and *P*-value < 0.05. Bioinformatics annotation was carried out using BLAST^+^ (version 2.9.0; National Center for Biotechnology Information, Bethesda, MD, USA) and InterProScan (version 5.0; European Bioinformatics Institute, Hinxton, UK). Data were visualized as heatmaps and volcano plots and subjected to PCA and Kyoto Encyclopedia of Genes and Genomes (KEGG) pathway enrichment analysis to elucidate underlying biological pathways.

### Data acquisition and analysis for glial fibrillary acidic protein and reticulon 4 in the human brain

GFAP and RTN4 expression data were derived from the Seattle Alzheimer’s Disease Cell Atlas (https://sea-ad.org) and the CZ CELLxGENE Discover data platform (https://cellxgene.cziscience.com/) (CZI Cell Science Program et al., 2025). The data were visualized using the ABC Atlas tool, which is part of the Allen Brain Cell Atlas (https://portal.brain-map.org/atlases-and-data/bkp/abc-atlas).

We selected a human brain dataset containing annotated cell types from both AD donors and healthy controls. Using the portal’s visualization tools, we queried GFAP and RTN4 expression. We then generated Uniform Manifold Approximation and Projection (UMAP) plots to display the cellular distribution of these genes. The primary outcome indicator was the visualized gene expression level within the annotated astroglia cell cluster, where color intensity directly corresponds to the normalized expression level. The resulting images were then exported for figure preparation. All procedures were performed in accordance with the guidelines provided on the websites for analysis and data download.

### Cell culture

The SVGP12 cell line (RRID: CVCL_3797), an immortalized human glial cell line, was sourced from Central South University (Changsha, China) and maintained in minimum essential medium (Hyclone) supplemented with 10% fetal bovine serum (Gibco, Waltham, MA, USA) and 1% penicillin-streptomycin. Cell cultures were kept in a humidified incubator at 37°C with 5% CO_2_. When the cells reached about 80%–90% confluence, they were passaged to maintain viability. An inverted microscope was used for regular monitoring of cell morphology and growth to ensure consistent cell quality and confluency.

### Cell viability and morphology

SVGP12 cell viability was assessed by plating 10,000 cells/well in 96-well plates containing 100 µL complete minimum essential medium per well. The cells were incubated overnight to allow them to adhere to the bottoms of the wells. Next, the cells were treated with lipopolysaccharide (LPS; 1 mg/mL; Sigma-Aldrich), an inflammatory stimulus, for 24 hours. Then, a Cell Counting Kit-8 (CCK-8; C6005, NCM Biotech, Suzhou, China) was used according to manufacturer’s protocol to measure cell viability: 10 µL of CCK-8 reagent was added directly to each well, and the plates were incubated for 1–2 hours at 37°C. Cell viability was determined by measuring the optical density at 450 nm (OD450) using a BioTek Epoch microplate reader (BioTek Instruments, Winooski, VT, USA).

SVGP12 cells were plated at 1 × 10^5^ cells/well in six-well plates. After culturing for 24 hours, the cells were treated with LPS. Cell morphology was evaluated using an optical microscope (Olympus CX23, Tokyo, Japan).

### Western blotting

After the experimental treatments had been applied, the cells were harvested by centrifuging at 1000 × *g* for 10 minutes at 4°C. The pellets were immediately homogenized in ice-cold radioimmunoprecipitation assay buffer (Sigma-Aldrich) plus protease/phosphatase inhibitors (Sigma-Aldrich) and incubated on ice for 5 minutes with periodic vortexing to ensure complete lysis. The lysates were cleared by centrifuging at 12,000 × *g* for 17 min at 4°C, and the resulting supernatants were collected and frozen at –80°C for later use. The protein concentrations were measured using a bicinchoninic acid protein assay kit in accordance with the manufacturer’s protocol. Subsequently, equivalent protein amounts (30 µg per lane) for each sample were separated by sodium dodecyl sulfate–polyacrylamide gel electrophoresis. Following electrophoresis, the proteins were transferred to polyvinylidene fluoride membranes for subsequent immunoblotting. The membranes were blocked with 5% nonfat milk/Tris-buffered saline with Tween 20 to prevent nonspecific antibody binding. Next, the membranes were incubated for 12–14 hours at 4°C with primary antibodies against RTN4 (rabbit polyclonal antibody, 1:1000, Proteintech, Cat# 10740-1-AP, RRID: AB_2183598), GFAP (mouse monoclonal antibody, 1:2000, Proteintech, Cat# 60190-1-Ig, RRID: AB_10838694), and glyceraldehyde-3-phosphate dehydrogenase (rabbit polyclonal antibody, 1:5000, Proteintech, Cat# 10494-1-AP, RRID: AB_2263076), with glyceraldehyde-3-phosphate dehydrogenase used as the loading reference. Then, the membranes were washed with Tris-buffered saline with Tween 20 and exposed to the appropriate secondary antibodies (horseradish peroxidase–conjugated goat anti-mouse IgG secondary antibody, 1:2000, Proteintech, Cat# SA00012-6, RRID:AB_2890969; horseradish peroxidase–conjugated goat anti-rabbit IgG secondary antibody, 1:1000, Proteintech, Cat# SA00001-2, RRID: AB_2722564) for 2 hours at 37°C. Excess secondary antibody was removed by washing the membranes three times with Tris-buffered saline with Tween 20, for 10 minutes each time. The protein bands were detected using an enhanced ECL chemiluminescence system (Bio-Rad, ChemiDoc, Hercules, CA, USA), and band intensities were measured with ImageJ software (v1.8.0, National Institutes of Health, Bethesda, MD, USA; Schneider et al., 2012).

### Statistical analysis

No statistical methods were used to predetermine sample sizes; however, our sample sizes are similar to those reported in a previous publication (Yi et al., 2020). All statistical analyses were performed using GraphPad Prism software (version 9.5.0 for Windows, GraphPad Software, San Diego, CA, USA, www.graphpad.com). Data are expressed as mean ± standard error of the mean (SEM). For comparisons between independent two groups, unpaired, two-tailed Student’s *t*-test was applied. For experiments involving more than two groups, two-way analysis of variance was conducted. *Post hoc* analyses were performed using Tukey’s multiple comparisons test. Statistical significance was defined as *P* < 0.05.

## Results

### Cognitive and pathological features of amyloid precursor protein/presenilin 1 mice

**Figure 1:**
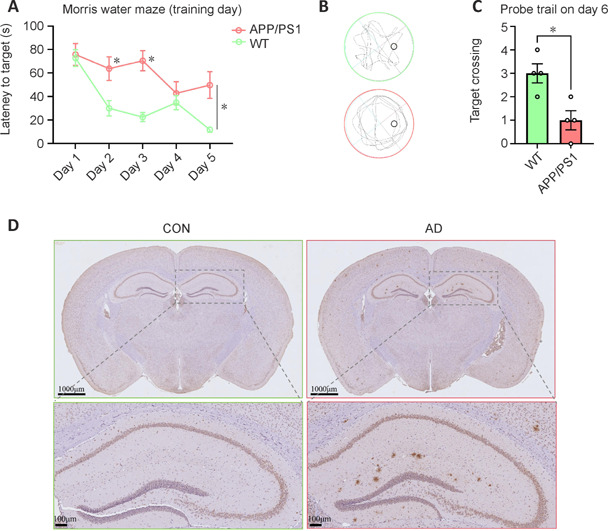
Cognitive dysfunction in APP/PS1 mice. (A) Escape latency during the MWM test. APP/PS1 mice exhibited significantly increased escape latency compared with WT mice, indicating impaired learning ability. (B) Representative swimming trajectories of WT and APP/PS1 mice during the probe test. APP/PS1 mice demonstrated less targeted search patterns than WT mice, suggesting reduced memory retention. (C) The number of platform crossings in the target quadrant in 1 minute. APP/PS1 mice crossed the platform location significantly fewer times than WT mice, reflecting memory deficits. Data are expressed as mean ± SEM (*n* = 4). *P < 0.05, *vs*. WT group (repeated measures two-way analysis of variance followed by Tukey’s test [A] and unpaired two-tailed *t*-test [C]). (D) Representative immunohistochemistry images of amyloid-beta (Aβ) plaque deposition in the brain. Significant Aβ plaque deposition was observed in the hippocampal region of the AD (APP/PS1) group, while no such deposition was detected in the CON (WT) group. Scale bars: 1000 µm (upper), 100 µm (lower). AD: Alzheimer’s disease; APP/PS1: amyloid precursor protein/presenilin 1; Aβ: amyloid beta; CON: control; MWM: Morris water maze; WT: wild type.

The MWM test was employed to evaluate spatial learning and memory abilities in the mice. During the escape latency phase (days 1–5), the APP/PS1 mice demonstrated significantly increased escape latencies compared with WT mice (**Figure 1A**). On day 6, the platform was removed to assess spatial memory. The WT mice displayed intricate and disordered swimming trajectories, while the APP/PS1 mice showed a consistent and direct swimming path. These results indicate that the APP/PS1 mice had impaired spatial exploration (**Figure 1B**). Moreover, the APP/PS1 mice exhibited fewer platform crossings than the WT mice, further supporting their spatial memory deficits (**Figure 1C**).

Immunohistochemistry was used to evaluate Aβ plaque deposition. As shown in **Figure 1D**, there was pronounced Aβ plaque deposition in the brains of APP/PS1 mice, but no such deposition was observed in the brains of WT mice. In summary, these results demonstrate that 9-month-old APP/PS1 mice exhibit pronounced cognitive impairment and Aβ plaque deposition.

### Transcriptomic and proteomic differences between wild type and amyloid precursor protein/presenilin 1 mice

**Figure 2:**
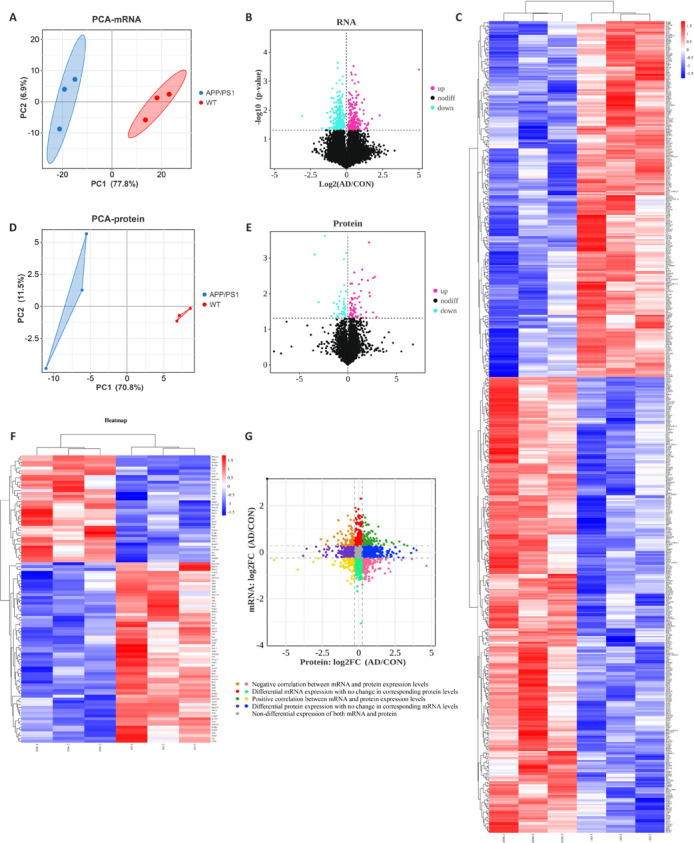
Transcriptomic and proteomic alterations in APP/PS1 mice. (A) PCA analysis of differentially expressed mRNAs between WT and APP/PS1 mice. (B) Volcano plot of differentially expressed mRNAs (*P* < 0.05, FC ≥ 1), with pink and blue dots representing upregulated and downregulated genes, respectively. (C) Heatmap of differentially expressed mRNAs, with red and purple indicating upregulated and downregulated mRNAs, respectively. (D) PCA of differentially expressed proteins between WT and APP/PS1 mice. (E) Volcano plot of differentially expressed proteins (*P* < 0.05, FC ≥ 1), with pink and blue dots representing upregulated and downregulated proteins, respectively. (F) Heatmap of differentially expressed proteins, with red and purple indicating upregulated and downregulated proteins, respectively. (G) Nine-quadrant map visually depicting the various relationships between mRNA and protein expression. APP/PS1: Amyloid precursor protein/presenilin 1; FC: fold change; PCA: principal component analysis; WT: wild type.

Next, RNA sequencing was performed to explore gene expression differences. First, a total of 30,405 mRNAs were identified. PCA of the mRNA data revealed a clear separation between the WT and APP/PS1 mice, indicating distinct gene expression profiles between the two groups (**Figure 2A**). In total, 555 genes were differentially expressed, with 240 upregulated and 315 downregulated in APP/PS1 mice compared with the WT group. The gene expression changes were visualized as heatmaps (**Figure 2B** and **C**).

To complement the transcriptomic analysis, proteomics analysis was performed to assess the brain proteome. A total of 35,683 unique peptides from 5588 proteins were identified. Differentially expressed proteins were defined using the thresholds of fold change ≥ 1 and *P*-value < 0.05. PCA of the proteomic data also revealed a clear distinction between the two groups (**Figure 2D**), consistent with the mRNA data. A total of 102 proteins with significantly altered expression levels were identified, with 64 upregulated and 38 downregulated in APP/PS1 mice compared with the WT group (**Figure 2E** and **F**).

Next, transcriptome–proteome association analysis was performed to explore the relationship between mRNA and protein expression (**Figure 2G**). The resulting nine-quadrant map revealed mRNA–protein pairs with a variety of negative and positive correlation patterns, including changes in mRNA levels that did not align with changes in protein levels, protein level changes without mRNA level changes, and similar levels of mRNA and protein expression (**Figure 2G**). These results imply that the expression of some genes may be regulated at the post-transcriptional or translational level.

### m6A methylation and phosphorylation modifications in amyloid precursor protein/presenilin 1 mice

**Figure 3:**
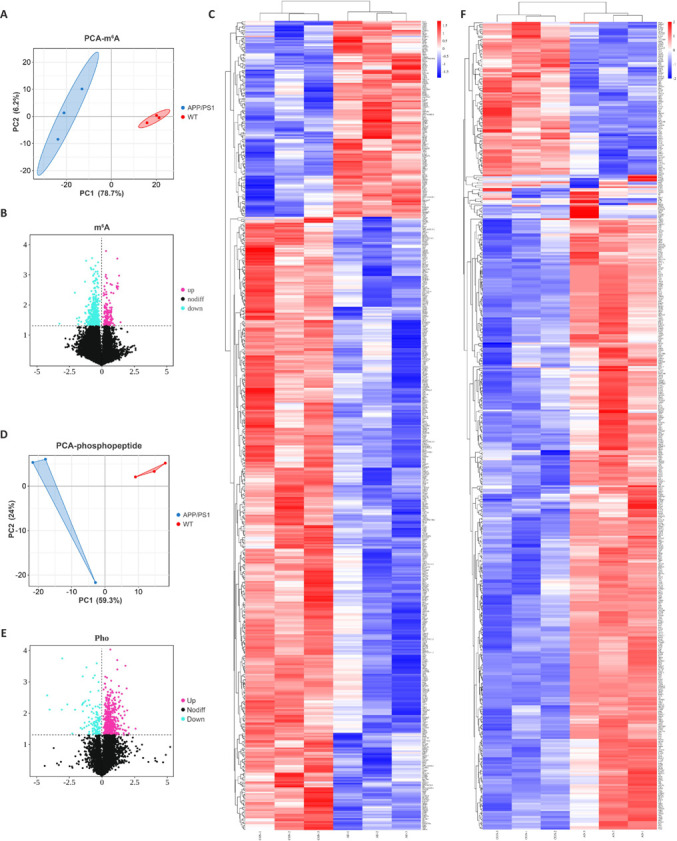
m^6^A methylation and phosphorylation modifications in APP/PS1 mice. (A) PCA of differentially m^6^A-modified mRNAs between WT and APP/PS1 mice. (B) Volcano plot showing differentially m^6^A-modified mRNAs (*P* < 0.05, FC ≥ 1), with pink and blue dots indicating hypermethylated and hypomethylated mRNAs, respectively. (C) Heatmap of differentially m^6^A-modified mRNAs, with red and purple indicating hypermethylated and hypomethylated mRNAs, respectively. (D) PCA of differentially phosphorylated peptides between WT and APP/PS1 mice. (E) Volcano plot showing differentially phosphorylated peptides (*P* < 0.05, FC ≥ 1), with pink and blue dots indicating upregulated and downregulated phosphorylated peptides, respectively. (F) Heatmap of differentially phosphorylated peptides, with red and purple indicating upregulated and downregulated peptides, respectively. APP/PS1: Amyloid precursor protein/presenilin 1; FC: fold change; m^6^A: N^6^-methyladenosine; PCA: principal component analysis; WT: wild type.

To investigate the possibility that some genes may be regulated at the post-transcriptional or translational level, we conducted MeRIP-Seq to identify genes with differential m6A methylation levels within the brains of WT mice compared with APP/PS1 mice. PCA of the differentially methylated genes revealed a clear separation between the two groups, highlighting pronounced m6A modification in the brains of APP/PS1 mice (**Figure 3A**). Differential analysis identified 528 differentially methylated genes (fold change > 1 and *P* < 0.05) in APP/PS1 mice compared with WT mice. Of these, 426 genes were hypermethylated, while 102 genes were hypomethylated (**Figure 3B** and **C**). These results indicate that m6A modification may influence gene expression, potentially contributing to the observed differences in mRNA and protein levels.

Additionally, given the significant impact of phosphorylation on protein function and activity, we conducted a phosphoproteomic analysis that identified 10,648 phosphopeptides, 16,620 phosphosites, and 3994 phosphoproteins. Differentially phosphorylated peptides were selected based on significant differences between WT and APP/PS1 mice, with the following thresholds: fold change > 1 and *P* < 0.05. The PCA results revealed clear segregation into two distinct clusters based on the phosphoproteome (**Figure 3D**), with 481 downregulated and 121 upregulated phosphopeptides (**Figure 3E** and **F**). These findings suggest that phosphorylation may also account for the observed discrepancies in mRNA and protein expression levels, as a previous study showed that protein phosphorylation influences protein expression (Wu et al., 2021).

### Comprehensive analysis of post-transcriptional and post-translational modifications in amyloid precursor protein/presenilin 1 mice

**Figure 4:**
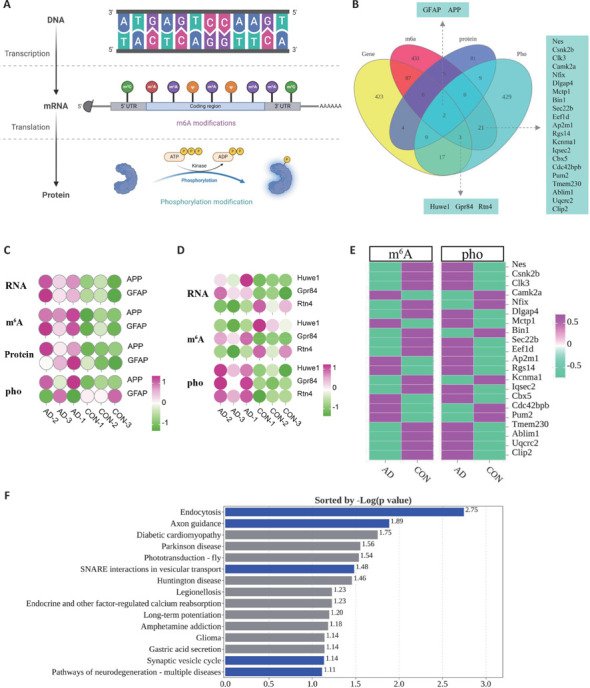
Multi-omics integration reveals key molecular pathways in APP/PS1 mice. (A) Schematic representation of the central dogma of molecular biology. (B) Venn diagram illustrating overlapping genes identified across four omics layers (mRNA expression, m^6^A methylation, protein phosphorylation, and protein expression), with detailed intersecting gene information highlighted in the light blue box. (C–E) Heatmaps displaying the expression levels of the intersecting genes across the different omics layers. (F) KEGG pathway enrichment analysis of the intersecting genes, with dark blue highlighting significantly enriched pathways. APP/PS1: Amyloid precursor protein/presenilin 1; KEGG: Kyoto Encyclopedia of Genes and Genomes; m^6^A: N^6^-methyladenosine.

To further explore the underlying mechanisms of AD, we conducted an integrated analysis encompassing transcriptomics, proteomics, m6A epitranscriptomics, and phosphoproteomics (**Figure 4A**). Venn diagram analysis identified two genes (GFAP and APP) whose mRNA, protein, methylation, and phosphorylation levels were all significantly altered (**Figure 4B** and **C**). Both GFAP and APP were expressed at notably higher levels in AD mice compared with WT mice, underscoring their critical roles in AD onset and progression. Furthermore, we identified three additional genes (*Huwe1*, *Gpr84*, and *Rtn4*) that were consistently altered at the mRNA, m^6^A, and phosphorylation levels (**Figure 4B** and **D**). Additionally, we detected 21 genes exhibiting significant differences in m^6^A modification and phosphorylation between the two groups, including *Nes*, *Csnk2b*, *Ck3*, *Camk2a*, *Nfix*, *Digap4*, *Metp1*, *Binl*, *Sec22b*, *Befld*, *Ap2mi*, *Resl4*, *Kenmal*, *Igsec2*, *Cbx5*, *Cdc42bpb*, *Pum2*, *Tmem230*, *Ablim1*, *Ugere2*, and *Clip2* (**Figure 4B** and **E**).

To better understand the biological significance of these findings, we performed KEGG pathway enrichment analysis. The results highlighted endocytosis as a potentially critical pathway in AD pathology (**Figure 4F**). Thus, we decided to focus our subsequent analyses on astrocytes and their contribution to AD.

### Elevated RTN4 expression in reactive astrocytes in Alzheimer’s disease

**Figure 5:**
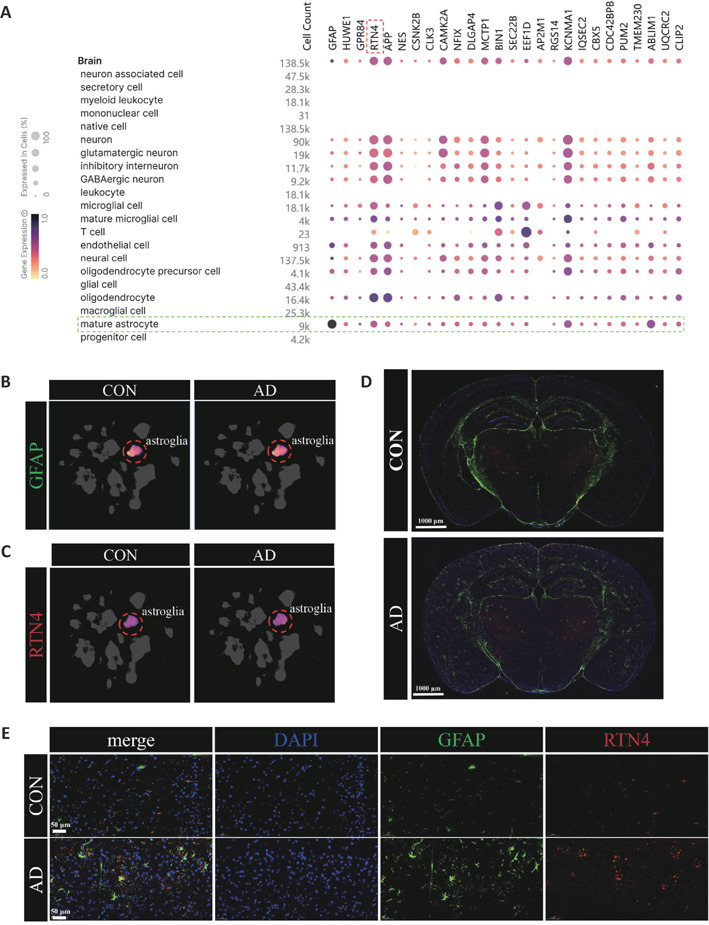
Elevated RTN4 expression in reactive astrocytes from APP/PS1 mice. (A) Heatmap of differentially expressed genes intersecting with public database data from the human brain, with darker colors indicating higher expression levels. (B, C) UMAP plots displaying GFAP and RTN4 expression, based on single-nucleus RNA sequencing data from the Seattle Alzheimer’s Disease Cell Atlas. Brighter colors represent higher expression levels, and red circles highlight astroglia. (D) Representative immunofluorescence images showing GFAP and RTN4 immunoreactivities in mouse brains. (E) Magnified immunofluorescence images of GFAP and RTN4 immunoreactivities in WT and APP/PS1 mouse brains. Compared with the CON group, APP/PS1 mice (AD group) exhibited a marked increase in GFAP-positive astrocytes, as indicated by enhanced green fluorescence intensity, and notable upregulation of RTN4 expression (red fluorescence) within these astrocytes. DAPI (blue): nuclear staining; GFAP (green, Alexa Fluor 488): astrocyte marker; RTN4 (red, Alexa Fluor 594): reticulon-4 protein. Scale bars: 50 µm. AD: Alzheimer’s disease; APP/PS1: amyloid precursor protein/presenilin 1; CON: control; DAPI: 4′,6-diamidino-2-phenylindole; GFAP: glial fibrillary acidic protein; RTN4: reticulon 4; UMAP: uniform manifold approximation and projection; WT: wild type.

To investigate how the differentially methylated, phosphorylated, and expressed genes that we identified regulate astrocyte function, we explored the relationship between these genes and astrocytes using public databases. We found that RTN4 expression was elevated in astrocytes from the brains of patients with AD, while that of other genes was not (**Figure 5A–C**). This finding was supported by immunofluorescence staining, which showed an increase in GFAP fluorescence intensity, as well as RTN4 upregulation, in GFAP-positive astrocytes in APP/PS1 mice compared with WT mice (**Figure 5D** and **E**). These results suggest that RTN4 may regulate astrocyte activation, thereby contributing to AD pathogenesis.

**Figure 6:**
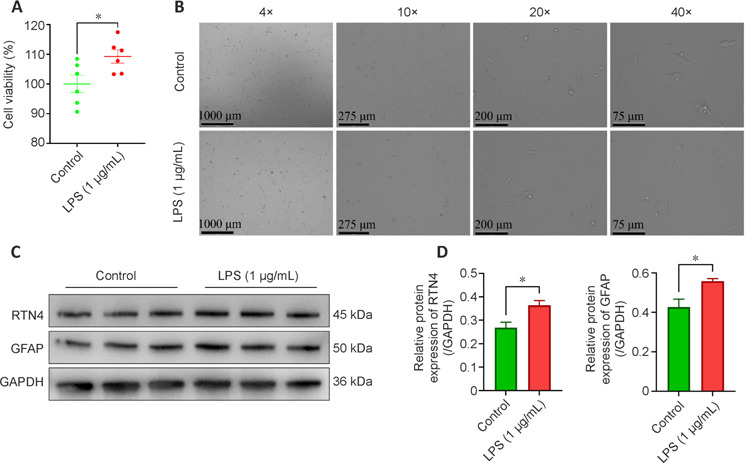
Validation of RTN4 expression in LPS-induced astrocytes. (A) CCK-8 assay showing astrocyte viability after LPS treatment (1 μg/mL). (B) Light microscopy images illustrating morphological changes in astrocytes following LPS treatment. Compared with the control group, astrocytes treated with LPS exhibited clear morphological changes, including enlarged cell bodies and an increased number and length of cellular processes. These features are indicative of astrocyte activation in response to inflammatory stimulation. (C) Western blot analysis of RTN4 and GFAP expression in astrocytes after LPS treatment. (D) Quantification of RTN4 and GFAP protein expression levels based on western blot analysis. Data are expressed as mean ± SEM (*n* = 3). *P < 0.05 (unpaired two-tailed *t*-test). CCK-8: Cell counting kit-8; GFAP: glial fibrillary acidic protein; LPS: lipopolysaccharide; RTN4: reticulon 4.

Next, we sought to validate these results *in vitro*. Therefore, we activated astrocytes with LPS. CCK-8 assay showed that LPS treatment significantly increased astrocyte activity (**Figure 6A**). Light microscopy assessment revealed noticeable morphological changes, including cell body enlargement and an increased number of processes, which are characteristic of activated astrocytes (**Figure 6B**). Furthermore, western blotting showed a significant increase in both GFAP and RTN4 expression in LPS-treated astrocytes (**Figure 6C** and **D**). These findings suggest that RTN4 contributes to AD pathogenesis by regulating astrocyte activation.

## Discussion

This study employed an integrated multi-omics approach to thoroughly investigate molecular alterations linked to AD in APP/PS1 transgenic mice. By combining transcriptomics, proteomics, m6A epitranscriptomics, and phosphoproteomics analyses, we identified important molecular changes associated with AD progression. Specifically, our findings highlighted several key genes, including GFAP and APP, whose differential expression across multiple omics platforms suggests that they play critical roles in AD pathology. Additionally, the significant increase in RTN4 expression within reactive astrocytes indicates its potential involvement in neuroinflammation, a hallmark of AD. The comprehensive multi-omics approach utilized in this study provides novel insights into AD mechanisms and identifies promising therapeutic targets, especially RTN4.

Increasing evidence indicates that reactive astrocytosis is central to the pathology of AD and other neurodegenerative diseases (Ding et al., 2021; Brandebura et al., 2023; Chiotis et al., 2023; Qian et al., 2023). A hallmark characteristic of these reactive astrocytes is upregulation of GFAP, a protein that is primarily expressed by astrocytes and is associated with alterations in the astrocyte cytoskeleton, including hypertrophy and process extension toward injured areas (Yang and Wang, 2015; Escartin et al., 2021). Consistent with previous studies (Kim et al., 2025; Yang et al., 2025), we found that astrocyte activation is associated with AD pathology. Astrocyte reactivity is now recognized as a hallmark of AD pathology, alongside plaques and tangles (Bassil et al., 2021; Price et al., 2021; Lee et al., 2022). We also observed morphological changes in astrocytes, which aligns with growing evidence that reactive astrocytes, identified by increased GFAP expression, actively contribute to neurodegeneration by clustering around amyloid plaques and enhancing neuroinflammation (Pereira et al., 2021; Ferrari-Souza et al., 2022; Chatterjee et al., 2023). Elevated plasma GFAP expression has been proposed as a potential early biomarker for AD due to its correlation with glial activation and disease severity (Johansson et al., 2023). Our findings align with previous studies that link GFAP upregulation to neuroinflammation (Pereira et al., 2021; Chatterjee et al., 2023). In AD, reactive astrocytes release proinflammatory cytokines, which exacerbate neurodegeneration by promoting Aβ production and impairing microglial Aβ clearance (Su et al., 2021; Phillips et al., 2024). This creates a positive feedback loop that may persist for years before the onset of clinical symptoms (Smit et al., 2021; Chandra et al., 2023). Therefore, therapeutic strategies targeting astrocyte activation pathways could offer novel strategies for modulating neuroinflammation in AD, as supported by previous studies demonstrating cognitive improvement following a reduction in astrocyte activation (Smit et al., 2021; Luo et al., 2024).

Another important finding from our mouse model is that RTN4 expression was increased in reactive astrocytes. RTN4 and other reticulon family proteins have been implicated in neurodegenerative diseases and neuroinflammation (Xiao et al., 2022). RTN4 is a neurite growth inhibitor, and its expression is linked to inhibition of vascularization in the developing central nervous system and postnatal brain (He et al., 2004; Wang et al., 2020). Moreover, Nogo-A, a protein encoded by the *Rtn4* gene, and its receptor NgR1 are overexpressed in the hippocampus of patients with AD (Pavon et al., 2023). Furthermore, recent studies have shown that Rtn4 knockdown promotes angiogenesis and improves spatial learning (Raiker et al., 2010; Xiao et al., 2022). Additionally, loss of RTN4 function in mice was associated with improvements in spatial learning, as well as synaptic plasticity in the hippocampus, which further supports the notion that inhibition of RTN4 may reverse cognitive impairments in AD (Zagrebelsky et al., 2017). Moreover, a recent study showed that inhibiting the Nogo-A/NgR1 pathway in APP/PS1 mice effectively restored synaptic plasticity and memory-associative long-term potentiation (Pavon et al., 2023). However, another study reported that post-stroke astrocytic Nogo-A upregulation may exert protective anti-inflammatory effects by restricting macrophage infiltration (Boghdadi et al., 2021), highlighting the complexity of RTN4 in neuropathology. Taken together, these studies suggest that RTN4 is a potential therapeutic target for modulating glial activity and enhancing cognitive function in AD (Rodriguez et al., 2022). Indeed, one study showed that targeting RTN4 improved neuronal function (Xiao et al., 2022). However, given the multiple physiological roles of Nogo-A in the nervous system and its expression in other cell types, inhibiting its activity may lead to various side effects. Future research should investigate these interactions and evaluate RTN4-targeted therapies, including small-molecule inhibitors or gene silencing, for their effectiveness in alleviating AD symptoms.

This study also highlights the importance of m^6^A RNA methylation and protein phosphorylation in AD. In particular, one study reported altered methylation and phosphorylation of genes such as Camk2a (which is important for synaptic plasticity, calcium homeostasis, and memory formation) (Martinez-Feduchi et al., 2024). Camk2a dysfunction has been closely linked with AD susceptibility and pathogenesis, making it a target for therapy (Martinez-Feduchi et al., 2024). A recent study showed that m^6^A modifications in the 3′ untranslated region of the *Camk2a* mRNA are essential for its proper localization to neurites (Flamand and Meyer, 2022), suggesting that these modifications could be pivotal for synaptic function and plasticity. Thus, regulating Camk2a methylation could represent a promising approach to restoring synaptic function and mitigating cognitive decline in AD.

In addition to these molecular insights, our multi-omics approach also showed that endocytosis-related processes are significantly enriched in AD. Endocytosis is impaired in AD, particularly in regions of the brain that are susceptible to neurodegeneration, and this could lead to reduced secretion of neurotransmitters (Sze et al., 2000; Yang et al., 2015; De Bastiani et al., 2023). Astrocytes have been implicated in maintaining synaptic strength and plasticity through astrocytic vesicular endocytosis of glutamate and adenosine triphosphate (Pham et al., 2021; Li et al., 2025c). Our findings suggest that impaired endocytosis in astrocytes leads to synaptic dysfunction in AD.

Additionally, dysregulation of mRNA (transcriptomic) and protein (proteomic) expression levels was observed in APP/PS1 mice compared with WT mice. Only partial overlap was found between differentially expressed transcripts and proteins, indicating significant divergence between the transcriptome and proteome. This aligns with a recent large-scale analysis of human AD brains demonstrating that many protein-level changes lack corresponding mRNA alterations (Zhang et al., 2025). A comprehensive study found that almost half of co-expression modules for AD-associated proteins are not present in transcriptomic networks from the same brains. In addition, the mitogen-activated protein kinase signaling pathway is a protein-specific network module that is unique to AD, but was undetectable in mRNA profiles, emphasizing the role of post-transcriptional regulation in AD (Johnson et al., 2022). Differential m^6^A methylation of specific mRNAs may explain the differences between transcript levels and protein levels because of decreased translation efficiency and/or increased mRNA stability. In keeping with this, APP/PS1 mice show elevated levels of global m^6^A modification in the cortex and hippocampus, as well as increased expression of the m6A “writer” enzyme METTL3 and lower levels of the m^6^A “eraser” enzyme FTO (Han et al., 2020). Furthermore, analysis of protein phosphorylation in AD revealed that, with some exceptions, it is an important regulatory mechanism involved in the pathogenesis of the disease. For example, the increased phosphorylation of GFAP at Thr-299 and the microglial marker PTPRC (CD45) at Ser-964 in AD models indicates that astrocytes and microglia, respectively, are hyperactivated in AD (Yarbro et al., 2025). The transcriptomic or total proteomic data alone do not show these regulatory events. In summary, post-transcriptional and post-translational modifications in AD interfere with RNA and protein expression, resulting in an imbalance in their levels. Therefore, comprehensive analysis integrating multiple omics layers is essential for a thorough understanding of AD pathogenesis.

While our findings shed light on the molecular mechanisms underlying AD, it is important to recognize a number of limitations associated with this study. First, although we identified key genes and proteins associated with AD, the *in vivo* functional relevance of these molecules still requires full validation. To understand the function of these molecules, future studies could investigate the functional consequences of targeting these molecules by employing techniques such as gene knockout, overexpression, or pharmacological inhibition. Animal studies should be performed to investigate which disease mechanisms are affected by RTN4 modulation, and whether this improves cognition. This step is essential for connecting molecular discovery and therapy. Second, our study was based on the APP/PS1 mouse model, a well-established transgenic model of AD. While this model is robust, it may not fully represent the diverse pathological features of human AD. Therefore, our findings need to be verified in various other AD models, such as models of tauopathy or sporadic AD, to have broader applicability. Furthermore, a notable limitation of the present study is its exclusive use of male mice. This decision was made to ensure consistency and enhance comparability with the extensive body of existing preclinical data in Alzheimer’s disease research, which has predominantly focused on male animals. This approach allowed for a focused, preliminary investigation within a well-established experimental framework. However, we fully acknowledge that this single-sex design precludes the assessment of potential sex-specific differences in the pathological mechanisms we investigated. Given that sex is a critical biological variable in AD, future studies are planned to include both male and female mice to address this limitation and provide a more comprehensive and generalizable understanding of our findings. Regarding our *in vitro* experiments, the main benefit of using the SVGP12 immortalized astrocyte cell line is its stable proliferation and reproducibility. Nonetheless, its immortalization may not fully replicate primary astrocyte characteristics or their interactions within the brain. Therefore, future studies using primary astrocytes or brain organoid models are recommended for greater translational relevance. Moreover, while we explored several important post-transcriptional modifications, including m^6^A and phosphorylation, the precise molecular mechanisms by which these modifications influence AD progression remain unclear. Future research should explore how these modifications influence RNA stability, translation efficiency, or protein functionality in the context of AD. Likewise, examining potential interactions between these modifications may uncover synergistic effects that amplify AD pathology, offering new therapeutic avenues. Additionally, we used LPS to activate SVGP12 astrocytes in our experiments to examine astrocyte responses in a controlled inflammatory environment; however, this model does not mimic a complex pathological microenvironment. In the future, we plan to establish 3D co-culture models or use primary astrocytes from an AD animal model to validate our findings in a more representative *in vitro* model of AD.

In conclusion, our integrative multi-omics approach offers comprehensive insights into the molecular complexities underlying AD. By identifying critical molecular targets such as GFAP, APP, and RTN, and emphasizing the significance of post-transcriptional and post-translational regulation in AD, our findings establish a basis for future therapeutic development to combat cognitive decline in AD. Nevertheless, further investigations are required to validate these targets and fully understand their therapeutic utility.

## Data Availability

*All data can be provided by the corresponding author upon reasonable request.*
